# Apples and oranges? The importance of recognizing heterogeneity in arts and health research publications

**DOI:** 10.3389/fpubh.2025.1670166

**Published:** 2025-09-24

**Authors:** Anita Jensen, Hilary Bungay, Nicola J. Holt

**Affiliations:** ^1^Center for Primary Health Care Research, Department of Clinical Sciences Malmõ, Lund University, Malmõ, Sweden; ^2^Nord University, Bodø, Norway; ^3^School of Allied Health & Social Care, Anglia Ruskin University, Chelmsford, United Kingdom; ^4^Health and Social Sciences, University of the West of England, Bristol, United Kingdom

**Keywords:** art and health, heterogeneity, arts-based activities, research, public health

## Introduction

Policymakers and practitioners often do not engage directly with academic research articles. Therefore, researchers must disseminate findings coherently and objectively. This includes transparent communication of the limitations of research and acknowledgment of diverse cultural contexts and factors. Recognizing the challenges related to applicability across different environments is essential, as a one-size-fits-all approach is rarely effective. In the field of arts and health, we encounter research studies making generic claims that arts engagement is beneficial for health and wellbeing. While measuring tools are described as part of the methodology, the studies often lack descriptions of specifically how, and which, arts discipline(s) are involved. Important aspects of the facilitation, context, access to activities and choices for participants are under-reported and under-researched (1). For example, Arts on Prescription (AoP) programmes often consist of various arts and culture activities combined with different options for participation (active vs. receptive). However, little is known about the content and delivery of the activities (2). Programmes are described as “arts for wellbeing,” “arts for health” or “arts and recovery,” without descriptions of the activities offered. Some reviews have also grouped together the arts as one classification [see: Fancourt and Finn (3), Curtis et al. (4), Jensen and Bonde (5)] and make claims of impact and effectiveness although the diagnoses of participants are also often unclear. Emphasizing that the arts are good for one's health, is as imprecise as stating “medicine” is good for your health. By using generic terms, we risk comparing apples with oranges.

We argue that detailing the actual arts activities (and facilitation) used in research studies, is imperative.

### What is meant by “the arts”? Disciplinary differences

Arts and health researchers are drawn from a broad spectrum of academic disciplines. Within the published literature they rarely define “arts” consistently, making comparison across these works, and knowledge synthesis, problematic (6). This reflects the long debate about what the “arts” in “arts and health” incorporates, compounded by differences across countries. For example, most pre-industrial societies do not have an independent overall concept of “art,” even though the population may engage in activities that those living in industrial societies may describe as the “arts” such as singing, painting and dancing (7). Similarly, in many non-Anglo-Saxon cultures (ranging from Indigenous communities in the Americas to classical traditions in Asia and Africa) creative expression is often inseparable from spirituality, ritual, or community life, reminding us that the Western category of “art” represents only one cultural framework rather than a universal standard (8). Exploring the terminology used from an educational perspective, Sonke et al. (9) posit that the use of the “arts” as plural indicates inclusion of visual, performing and literary arts, whereas the use of the singular “art” indicates only the visual arts. The term “creative arts” is also used which Sonke et al. suggests adopts a hierarchical view—the “fine arts” vs. “creative arts” or “crafts.”

Currently, the “arts” may be defined by genre or sector, (non-profit, commercial or government), where the activity takes place (e.g., community arts), whether they are undertaken alone or with others in a group (e.g., participatory arts), the mode of participation, active art-making, volunteering or audience participation, and whether they are live, recorded or on-line (10). Parkinson (11) uses the term the “arts” to encompass everything, including the skills of the artist to the passion of the amateur, creativity, culture, and heritage. It may not be a problem for researchers to use “the arts” as a metonym for all the activities which may be considered under the umbrella term “arts,” except when reporting research findings where it is crucial that for clarity and consistency the reader knows specifically which of the different art forms are the subject of the research.

### The use of different terms: “the arts for wellbeing,” “arts for health” or “arts and recovery”

There is also a lack of consensus regarding the appropriate way to categorize the arts practices which impact on human, health, and wellbeing. The arts and health field is diverse, incorporating many different art forms and practices, in a range of healthcare, community and educational settings, which may be designed for specific health issues and used as primary, secondary and tertiary interventions. Parkinson (11) describes this as a “rich messy ecology,” preoccupied with humanizing clinical environments, targeting specific health outcomes and promoting cultural participation. Previously, White (12) identified five subtle permutations of terminology; “arts in health,” “arts for health,” “arts into health,” “arts and health,” and “healing arts,” each with different emphases, different approaches and different beliefs about health, ill-health and the place of arts practice in promoting health (and based on different epistemological underpinnings including biomedical, behavioral, community-based and social models). Fancourt (13) speculated that the phrase “arts and health” suggests that they are given equal weight (a common perspective in community-based models), whereas “arts in health” suggests that the arts are a way of supporting health in healthcare systems (sometimes associated with a biomedical perspective). The use of arts activities as interventions in healthcare may result in “arts and health” being governed by the health services and being reconceptualized as therapy or treatment (14). This then leads to yet another categorization, “arts as treatment” which is typically delivered by arts therapists with different status in different countries, for example in the UK they are state registered allied health professionals, using the arts in conjunction with other therapeutic approaches.

More recently, in the UK the term “creative health” was adopted by the National Center for Creative Health, to encapsulate all the arts and cultural activities within community and health care settings, and with individuals and community groups. Others, prefer to retain the use of the word “arts” on the basis that this acknowledges the role of arts practices, artists, arts policies and the arts sector in the development and delivery of arts and health (15). The on-going debate supports Broderick's contention that arts and health is subjective and a shifting amorphous entity (16). However, the main point here is to reiterate the heterogeneity of practice, and different theoretical lenses within the arts and health field, which it is important to specify in research reports.

### Lack of operationalization of the arts activities in research papers

Reviews of AoP programmes and other arts interventions for mental health have highlighted that many studies lack clear descriptions of the specific arts activities involved and often include diverse activities within a single study (2, 5). Other reviews have noted a similar lack of detailed protocols limiting the ability to evaluate, replicate and adapt interventions across contexts, or isolate which arts elements influenced outcomes [see Adams and Stickley; Havsteen-Franklin et al.; Zarobe and Bungay (17–19)].

This consistent lack of operationalization of what arts-based activities/ interventions are provided in practice means that we do not always know what they consist of and whether/why some work better than others. This gap hampers evaluation of the distinct impacts of various art forms and obstructs development of standardized interventions. In future, research unpicking and describing the various arts approaches is essential. Reporting of specific details of programme delivery (e.g., specific art activities, programme duration, facilitator style and skills, proposed mechanisms of action) rather than vague programme details (e.g., “a programme of weekly arts activities”) can help to build evidence for what works for whom and why.

### Lack of description of arts facilitation in research papers

Art activities/interventions are not directly comparable if we do not know what participants are engaging with, and what skills and strategies are used to support them in those interventions. In addition to variations in the art activities, how the art activity is facilitated can vary. Facilitation may be essential for any health and wellbeing impact, given that the “therapeutic alliance” developed with artist facilitators may additionally support wellbeing (20, 21). For example, the ways in which “safe spaces” are created by facilitators, through managing interpersonal and group dynamics, and encouragement of participants to be autonomous and creative with making, may be crucial to the success of art interventions (22). Skills and practices may vary according to art forms and outcomes and may be done in ways which are more or less impactful for wellbeing. However, the approach and method of facilitators is little understood or shared in research papers (23).This poses numerous problems, including for replicating effects and developing consistent good practice (2, 24). Such factors have well-documented impacts in other fields, such as psychotherapy, occupational, sports and health psychology and creativity studies (25–27), and have been found to moderate outcomes in meta-analyses of interventions in other fields (28).

In addition to the lack of reportage of how engagement with the arts is facilitated, there is insufficient clarity about the amount of training and support practitioners have, whether they working as an “artist” or an “arts for health facilitator,” whether they been trained in safeguarding, ethics, pedagogy, communication or leadership (29). This is essential to meet the requirement that interventions “do no harm” and feed into future (30, 31). It is crucial not only to develop protocols about minimum training standards, ethics and guidelines, but also to report these (and facilitation methods) in research papers.

### Heterogeneity is a strength: what works best for whom, and how

We have discussed how arts interventions are extremely varied, from singing in a choir, crocheting in a group, mindful doodling “or Zentangling,” to dance interventions, and may be delivered with different goals, populations and facilitator styles. A further reason for specificity in the reporting and development of art activities/interventions relates to targeting the required outcomes for participants (32), specificity can help to determine what works best for whom, how and why.

For health systems to integrate arts for health consistently and effectively, and in a meaningful way, identifying the merits of specific arts programmes is required, rather than looking at “art and health” or “creative health” activities/interventions broadly. This would enable arts activities/interventions to be delivered in targeted ways to meet a range of health and wellbeing needs. For example, if we know attentional focus helps to reduce the perception of chronic pain, we can develop art interventions drawing on theoretical models about the conditions for absorbed attentional states. Such targeting is illustrated by work identifying the technical features of “singing for lung health,” rather than singing for wellbeing interventions, requiring songs with a specific breathing pattern and delivery for efficacy (33).

Reporting on and identifying mechanisms of action is essential for the development of theory, which is also lacking in the arts and health (23). Although work has been done to identify and systemise “active ingredients” (34), again, there is a focus on the field as a single unity, identifying hundreds of potential factors to consider. More focus on the “apples” and “oranges” and the specific benefits of the different arts activities/interventions is needed.

## Discussion

Arts and health is a heterogeneous field, with diversity in populations, interventions, disciplines, outcomes, and methodologies. In this Opinion piece, we have focused on the dangers of being non-specific when describing arts activities/interventions. While presenting these observations, we should also acknowledge that we (the authors) are not exempt from critique, as we have at times failed to provide detailed descriptions of the arts activities/interventions employed in our research. But “arts and health” is a generic term and recognizing the diversity of its offerings is essential for the field to develop further.

Specificity is crucial for multiple reasons, firstly so we are not comparing “apples with oranges” when considering efficacy, so we can meaningfully compare similar studies, and can improve meta-analyses and other reviews by enabling examination of which factors predict outcomes in heterogeneous datasets. Secondly, to have a better understanding of what art activities/ interventions consist of, enabling the replication findings and development of good practice. Thirdly, to develop understanding of “active ingredients” and mechanisms, led by and feeding into theory and practice, including the development of targeted art-based activities/intervention for specific outcomes. Finally, non-specificity can undermine policymaking, programme funding, and implementation.

Although challenging, due to the complexity involved in arts-based activities/interventions, we recommend that future published research describes the content, theoretical lens, facilitation methods (and training) and context of art-based activities/interventions in sufficient detail to meet these goals and develop the field (23). Arts-based activities and interventions are diverse in nature—a characteristic that offers both strength and adaptability. When delivered with the appropriate intensity (“dose”), facilitated effectively, and implemented in a suitable setting, they have the potential to enhance health and wellbeing.
